# Response to Tsuda et al.: the glomerular filtration rate is both age and allometrically influenced

**DOI:** 10.1038/s41440-025-02208-w

**Published:** 2025-05-09

**Authors:** Robert B. Rucker

**Affiliations:** Nutrition Department (AES) & Biochemistry (Medicine), University of California, Davis, CA USA

**Keywords:** Glomerular filtration rate (GFR), Allometric scaling

**Fig. 1:**
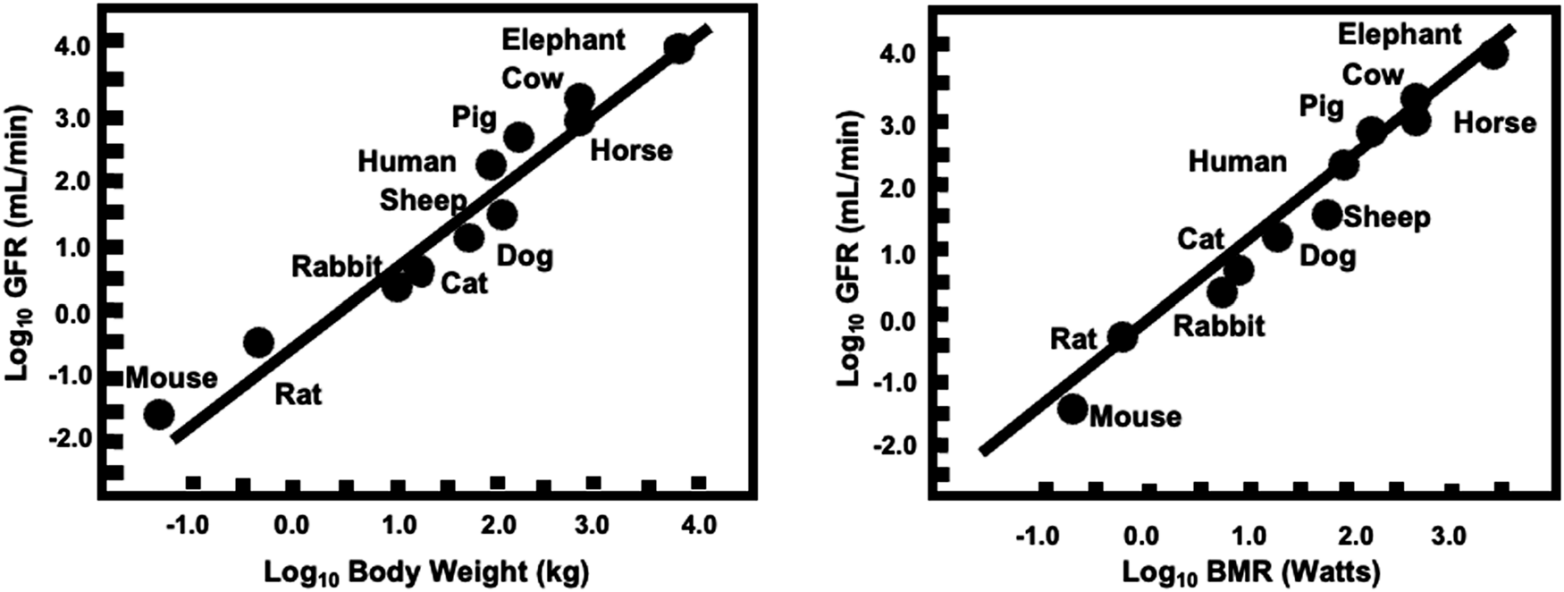
The logarithm of the Glomerular Filtration Rate (GFR) (mL/min) is plotted as a function of the log body weight (kg) and log Basal Metabolic Rate (BMR in Watts). The data were obtained from PubMed and Google Scholar searches, which conformed to the principles for biomedical research involving animals (e.g., http://grants.nih.gov/grants/olaw/olaw.htm). The relationships follow Kleiber’s Law, demonstrating that GFR increases allometrically with body mass and metabolic rate over a broad range of species [2]

Tsuda et al. [1] suggest that estimates of glomerular filtration rates (GFR) in renal assessments are compromised when allometric adjustments are made, such as corrections for surface area. Instead, they suggest that hyperfiltration in the context of GFR is best defined with age as the only adjustment. However, the GRF is a well-accepted allometric trait, and as shown in Fig. 1, GRF values from a wide range of species are linked inextricably to basal metabolism.

Moreover, when such relationships are ignored, endpoints important to growth, development, nutritional requirements, toxicological concerns, organ size, and aging can be easily misinterpreted [2, 3]. As an example, it is routine to compare the resting metabolic rate (RMR) of obese individuals to those of “normal or reference” individuals of the same height. Using isometric projections, the estimated BMR or resting metabolic weight (RMR) for 100–150 kg individuals can be ~1.7 to ~2.5 fold higher depending on the reference source. However, using allometric approaches (W2/3 or W3/4 power comparisons), the BMR or RMR for 100–150 kg individuals is substantially less. i.e., ~1.4 to 2-fold higher. Allometric scaling of resting energy expenditure removes body composition-associated biases and should always be considered in obesity and weight-based intervention studies [3]. Likewise, it is noteworthy that allometric scaling results in more precise results, even when applied to behavioral, cultural, and survey data. For example, in weight-stable adults, it is often reported that obese individuals often underreport their dietary intake to a greater extent than nonobese individuals. When allometric adjustments in body weight are made, this is not the case [4].

Although some agree that maximizing accuracy and uniformity in selecting one of the currently available GFR equations is difficult, those who have tried consistently use allometric approaches (mostly surface area comparisons). Rather than surface area, I prefer algorithms incorporating age and body weight as variables, such as a Kleiber modification of the Harris and Benedict equation [5]. It considers weight, age, and specific stature, which is defined as height in centimeters divided by weight to the 1/3 power (*S* = Height/*W*^1/3^).

$${{{\rm{BMR}}}}=	 \sim 70\times {{{\rm{weight}}}}\; {{{\rm{in}}}}\,{{{{\rm{kg}}}}}^{3/4}\times [1+0.004\,(30\!-\!{{{\rm{age}}}})\\ 	 +0.010\,({{{\rm{S}}}}\!-\!43.4)]$$

When applied to a typical subject in the study by Tsuda et al. [1], a 60-year-old person weighing ~80 kgs and height of 175 cm, the adjusted BMR or GFR is about 15 percent lower than a 30-year-old person of the same height and weight. Allometric scaling lowers five presumed abnormal values at or below the GFR-125/min threshold (data presented in the Graphical Abstract [1]).

In summary, physical components that influence basal metabolism and work are linked to renal functions. Whether viewed as a function of metabolic size (e.g., -kW3/4) or body surface area, GFR values are influenced by body weight consistent with biological laws that reflect allometric rather than isometric principles. Such principles should not be ignored, given that inflated GFR values may lead to unnecessary medicalization, overtreatment with medications, or overly restrictive diets that may cause harm [5].
